# Trichomicin Suppresses Colorectal Cancer *via* Comprehensive Regulation of IL-6 and TNFα in Tumor Cells, TAMs, and CAFs

**DOI:** 10.3389/fphar.2020.00386

**Published:** 2020-04-03

**Authors:** Xi Zhao, Xiaoqiang Qi, Wenrui Lian, Xin Tong, Hua Wang, Liya Su, Ping Wei, Zhuochen Zhuang, Jianhua Gong, Liping Bai

**Affiliations:** ^1^ NHC Key Laboratory of Biotechnology of Antibiotics, Institute of Medicinal Biotechnology, Chinese Academy of Medical Sciences and Peking Union Medical College, Beijing, China; ^2^ Department of Surgery and Ellis Fischel Cancer Center, University of Missouri-Columbia, Columbia, MO, United States; ^3^ Department of Gastrointestinal Surgery & Clinical Medicine Research Center, The Affiliated Hospital of Inner Mongolia Medical University, Hohhot, China; ^4^ Department of Medical Immunology, Basic Medical College, Shandong First Medical University and Shandong Academy of Medical Sciences, Taian, China

**Keywords:** Trichomicin, antitumor, colorectal cancer, signal transducer and activator of transcription 3, programmed cell death ligand 1, nuclear factor κB

## Abstract

Trichomicin, a small-molecule compound isolated from fungi, has been identified with bioactivity of antitumor. In this study, a colon cancer subcutaneous mice model was used to evaluate the antitumor effects of Trichomicin *in vivo*. Treatment with Trichomicin significantly inhibited tumor growth in a xenograft mouse colon cancer model. The underlying molecular mechanism has also been investigated through the quantification of relevant proteins. The expression levels of IL-6 and TNFα were reduced in tumor tissues of mice treated with Trichomicin, which was consistent with results of *in vitro* experiments in which Trichomicin suppressed the expression of IL-6 and TNFα in tumor and stromal cells. In addition, Trichomicin inhibited TNFα-induced activation of NF-κB and basal Stat3 signaling *in vitro*, which resulted in reduced expression of the immune checkpoint protein PD-L1 in tumor and stromal cells. Conclusively, Trichomicin, a promising new drug candidate with antitumor activity, exerted antitumor effects against colon cancer through inhibition of the IL-6 and TNFα signaling pathways.

**Graphical Abstract:**
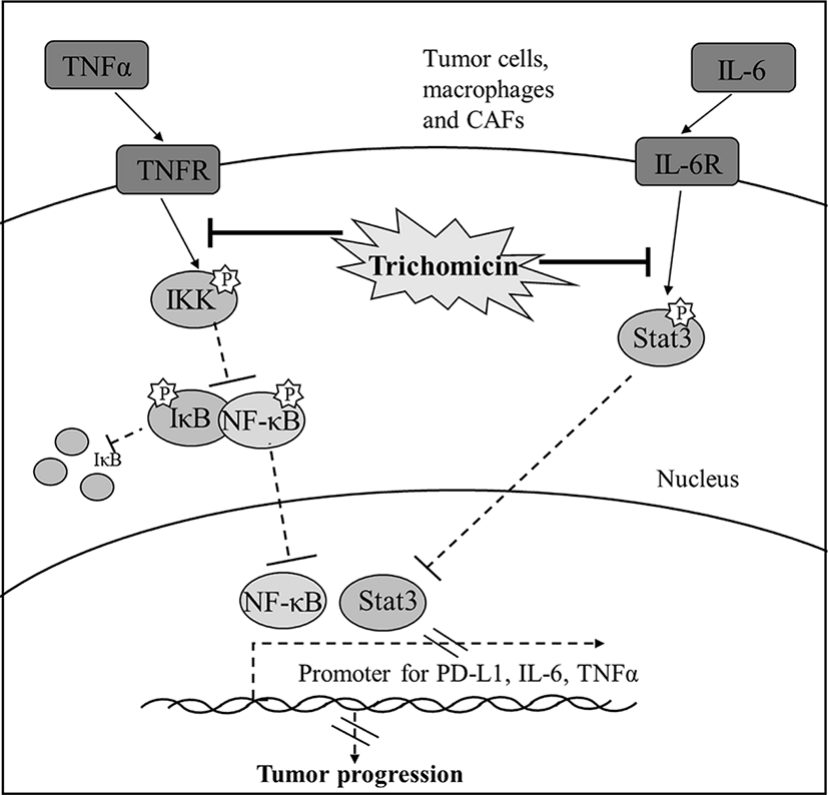
Diagram illustrating the signaling cascade that Trichomicin regulates TNFα-NF-κB and IL-6-Stat3 pathways.

## Introduction

Colorectal cancer (CRC) is the 3^rd^ most common cancer and the 2^nd^ leading cause of cancer-related death worldwide, according to WHO data in 2019. Furthermore, the incidence of CRC in individuals younger than 50 years of age has increased (Ganesh et al., 2019). The combination of chemotherapy and anti-angiogenic therapy is the first line treatment for metastatic colorectal cancer (mCRC). Unfortunately, drug-resistance is a major cause of poor survival rates in patients with CRC (Van der Jeught et al., 2018; Dekker et al., 2019). Immune checkpoint therapy was approved in 2017 for treatment of severely mutated CRC with high levels of microsatellite instability (MSI-H), but its efficacy is strongly associated with cancer metabolism and the tumor microenvironment (TME) (Pedrosa et al., 2019). The TME contains immune cells, cancer cells, and surrounding stromal cells, which are important sources of cytokines that can promote development and progression of CRC (Koumenis et al., 2014; Zitvogel et al., 2017). Therefore, modulation of the TME may inhibit tumor growth or maximize immunotherapeutic benefits in patients with CRC (Eyob et al., 2013; Adams et al., 2015).

Some cytokines in the TME promote cancer cell proliferation and tumor growth, particularly in CRC (Crusz and Balkwill, 2015; West et al., 2015). In the dextran sodium sulfate-azoxymethane (DSS-AOM) model, IL-6-dependent Stat3 signaling is a critical promoter of CRC cell survival and proliferation (Bollrath et al., 2009; Grivennikov et al., 2009). Although TNFα was first identified as a tumor-suppressive cytokine, it has been shown to promote tumors through increased inflammation in CRC (Popivanova et al., 2008; Balkwill, 2009). Increased levels of TNFα and IL-6 were detected in tumor tissues and sera of patients with colorectal cancer, and these cytokines were associated with increased tumor burden and tumor staging, metastasis, and lower overall survival (Coskun et al., 2017; Miranda et al., 2018). In addition, TNFα and IL-6 have been shown to promote activation of the key oncogenic transcription factors NF-κB (Nuclear factor κB) and Stat3 (Signal transducer and activator of transcription 3), which contribute to development and progression of CRC (Grivennikov and Karin, 2010; De Simone et al., 2015).

Trichomicin, a compound with antitumor activity, has been reported to significantly induce apoptosis in HT29 cells, and reduced IL-6 expression and phosphorylation of Stat3 in our previous study (Zhu et al., 2020). Based on these findings, we evaluated the antitumor effects of Trichomicin against colon cancer in a colorectal tumor animal model, and evaluated the role IL-6 and TNFα in these effects. Our results showed that Trichomicin significantly suppressed tumor activity in a CRC xenograft model by downregulating the expression of IL-6 and TNFα, which resulted in inhibition of the expression of the immune check point ligand protein, PD-L1, through inhibition of the Stat3 and NF-κB signaling pathways. Our findings provide a foundation for development of Trichomicin as an agent for treatment of CRC.

## Materials and Methods

### Cell Lines, Cell Culture, and Reagents

Human CRC cell lines HCT-116, Caco-2, and HT-29 and an acute monocytic leukemia cell line THP1 were obtained from China Infrastructure of Cell Line Resource (Beijing, China). All cell lines were verified using short tandem repeat (STR) analysis. Human colon carcinoma-associated fibroblasts (CAFs) were a gift from the affiliated hospital of Inner Mongolia Medical University and were identified by AmpFℓSTR Identifiler Plus PCR Amplification Kit prior to use. Cells were maintained in DMEM/F12 (CAFs), MEM (Caco-2), or RPMI 1640 (other cell lines) medium supplemented with 10% fetal bovine serum (FBS), 1% sodium pyruvate solution, and 1% penicillin/streptomycin (Gibco, Grand Island, NY, USA) in a 37°C, 5% CO_2_, fully humidified incubator. Trichomicin (1mM and 10mM, lab stock), PMA (10mM, Calbiochem, Darmstadt, GER), and BP-1-102 (5 mM, Selleck Chemicals, TX, USA) were dissolved in DMSO and were further diluted with medium (for cell assays) (Zhu et al., 2020). Interleukin 6 (10 μg/ml, Peprotech, NJ, USA), TNFα (10 μg/ml, Peprotech, NJ, USA), and lipopolysaccharide (LPS) (1 mg/ml, Calbiochem, Darmstadt, GER) were dissolved in PBS, passed through a 0.22–μm filter, then stored at -80°C (Zhu et al., 2020).

### *In Vivo* Xenograft Studies

Female nude mice (BALB/c, nu/nu, 18–22 g) were purchased from Beijing Vital River Laboratory Animal Technology. All animal experiments were approved by the Peking Union Medical College (PUMC) Pharmaceutical Institutional Animal Care and Use Committee. A xenograft mouse model was generated by subcutaneously injecting HT-29 cells (1×10^7^ cells per mouse) into the flanks of 8-week-old female nude mice (eight mice per group, 24 mice in total). When tumor volumes reached 200 mm^3^, Trichomicin was diluted in 0.5% (w/v) sodium carboxymethylcellulose (CMC), and administered at 60 mg/kg daily by oral gavage. Cisplatin (3 mg/kg) was administered intraperitoneally (I.P.) two times per week, and control animals received oral 0.5% (w/v) CMC and an I.P. injection that contained 100 μl of PBS as placebo. Tumor volume and body weight were measured twice per week. Following treatment, the mice were sacrificed and tumor tissues were processed for detection of IL-6/TNFα using enzyme-linked immunosorbent assay (ELISA).

### Quantification of IL-6/TNFα

Culture media from CRC cells were collected by centrifuging the cells at 1,000 rpm for 10 min following 48 h of cell culture. To generate macrophage-like cells, THP1 cells were incubated with 160 nM PMA at 37°C for 24 h at 1×10^5^ cells/well in 24-well plates (Zhu et al., 2020). Then cells were incubated with Trichomicin and LPS (1 μg/ml) or culture supernatants from different CRC cell lines (12.5%) in fresh culture medium for 12 h. The supernatants were collected and analyzed to determine cytokine profiles using ELISA (Boster, Wuhan, China). Total RNA extraction and cDNA synthesis were performed using an E.Z.N.A. HP Total RNA Kit (Omega Bio-tek, Doraville, GA, USA) and First-Strand cDNA Synthesis SuperMix (Transgen Biotech, Beijing, China) (Zhu et al., 2020). Quantitative real-time PCR was performed on a CFX96 Touch Real-Time PCR Detection System (Bio-Rad, CA, USA) using FastStart Universal SYBR Green Master (Roche, CA, USA) (Zhu et al., 2020). For each sample, the mRNA levels of IL-6 and TNFα were normalized to GAPDH levels and quantitated using the 2^−ΔΔCt^ method. Following with the primers used for real-time PCR: GAPDH (forward, 5′-GCACCGTCAAGGCTGAGAAC-3′; reverse, 5′-TGGTGAAGACGCCAGTGGA-3′), IL-6 (forward, 5′-CCACACAGACAGCCACTCAC-3′; reverse, 5′-TTTCACCAGGCAAGTCTCCT-3′) (Zhu et al., 2020), TNFα (forward, 5′-TGTAGCAAACCCTCAAGCTG-3′; reverse, 5′-TTGATGGCAGAGAGGAGGTT-3′).

### Gene Silencing Using RNAi Transfection

SignalSilence^®^ NF-κB p65 siRNA and control siRNA were obtained from Cell Signaling Technology (MA, USA). Lipofectamine RNAiMAX transfection reagent was used to transfect siRNA (Invitrogen, CA, USA). HCT-116 cells were seeded in 6-well plates (3×10^5^ cells/well) and allowed to adhere overnight. Then, NF-κB p65 siRNA or control siRNA (25 pmol/well) was mixed with RNAiMAX reagent at a 1:3 ratio, and transfection was performed according to the manufacturer's instructions. The medium was removed after 3 days.

### Western Blot

Cell lysates were prepared in RIPA lysis buffer containing phosphatase inhibitor (Applygen Technologies, Beijing, China) as previously described (Zhang et al., 2016). Equal amounts of protein were separating using SDS-PAGE, then transferred to PVDF membranes (Millipore, MA, USA). After blocking, the membranes were incubated with the following primary antibodies at 4 °C overnight: Phospho-NF-κB p65 (Ser536), Phospho-Stat3 (Tyr705), Phospho-IKKα/β (Ser176/180), Phospho-IκBα (Ser32), IKKβ, Stat3, NF-κB p65, IκBα, PD-L1, and GAPDH (Cell Signaling Technology, MA, USA). Proteins were detected using HRP-conjugated secondary antibodies and Immobilon™ Western Chemiluminescent HRP substrate (Millipore, MA, USA). Protein band density was quantified using a ChemiDoc ™ Imaging System (Bio-Rad, CA, USA).

### Statistical Analysis

All quantitative results were obtained from at least three independent experiments, and are presented as the mean±SD. Differences between two groups were evaluated using Student's *t*-tests. P < 0.05 was considered statistically significant.

## Results

### Trichomicin Inhibited Tumor Growth and Secretion of IL-6 and TNFα *In Vivo*

**Figure 1:**
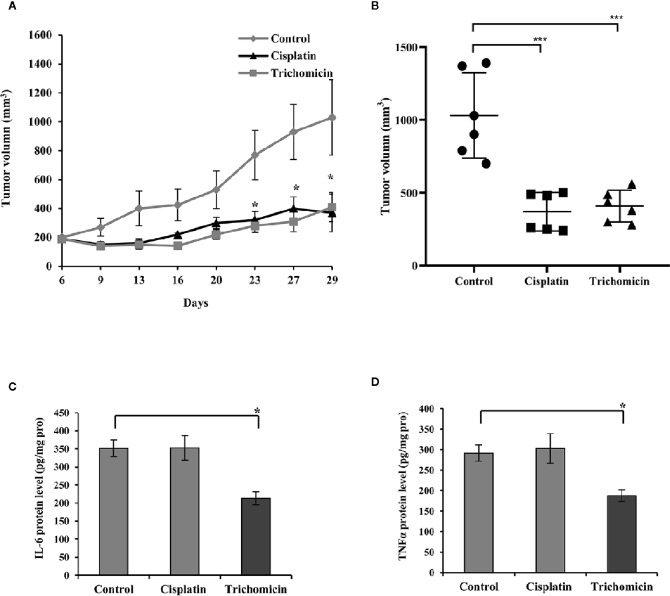
Trichomicin inhibited tumor growth and secretion of IL-6 and TNFα in an HT-29 tumor xenograft model. **(A)** Tumor growth curves for groups treated with vehicle, Trichomicin, or Cisplatin (n=6–7). ◆: Control. ▲: Cisplatin. ■: Trichomicin.: Trichomicin. **(B)** Comparison of the tumor volume measured on day 29 following the treatment of vehicle, Cisplatin and Trichomicin, respectively (n=6–7). ***P < 0.0005. ●: Control. ■: Cisplatin. ▲: Trichomicin. **(C, D)** Detection of IL-6 **(C)** and TNFα **(D)** levels in tumor tissues. Data are presented as the mean±SD, *P < 0.05, compared with the control group.

We evaluated the antitumor activity of Trichomicin in a nude mouse xenograft model generated by inoculation with HT-29 cells. Mice were treated with Trichomicin, Cisplatin, and vehicle, as previously described Transport assay (**Figure S1**), showed that Trichomicin was able to permeate across Caco-2 monolayers, and was administered orally in this study. As shown in **Figures 1A, B**, Trichomicin significantly reduced tumor volume compared to the control group (P < 0.05), and Trichomicin significantly inhibited tumor growth to a similar degree as cisplatin, with inhibitory rates of 62.70% and 61.88%, respectively. None of the treatments induced body weight loss or changes in behavior during this study.

The levels of IL-6 and TNFα in tumor tissues were quantitated using ELISA. The levels of IL-6 and TNFα decreased by 30.8% and 35.6% compared with those in the control group following treatment with Trichomicin (**Figures 1C, D**; p < 0.05), and cisplatin did not alter IL-6 and TNFα levels. These results suggested that the Trichomicin has a distinct mechanism with that in cisplatin to inhibit the growth of colorectal cancer, and the reduced expression of TNFα and IL-6 may be involved.

A preliminary pharmacokinetic study was performed in rats administered 200 mg/kg of Trichomicin. Trichomicin was detected in plasma 30 min post-administration (**Figure S2**), and reached a maximum plasma concentration (Cmax) of 4.8 μM 4 h after oral administration. The elimination half-life (T_1/2_) was 4.85 ± 0.4 h.

### Trichomicin Inhibited the Expression of IL-6 and TNFα in Macrophages

Tumor-associated macrophages (TAMs) are a major component of tumor immune cell infiltrates, and are a major source of IL-6 and TNFα in the TME. Cytokines such as IL-6 and TNFα are central players in CRC, and drive activation of signal transducer and activator of transcription 3 (Stat3), and a transcription factor nuclear factor-κB (NF-κB). To further characterize the antitumor mechanism of Trichomicin in CRC, we investigated the effects of Trichomicin on the expression and secretion of IL-6 and TNFα in THP1 macrophages.

**Figure 2:**
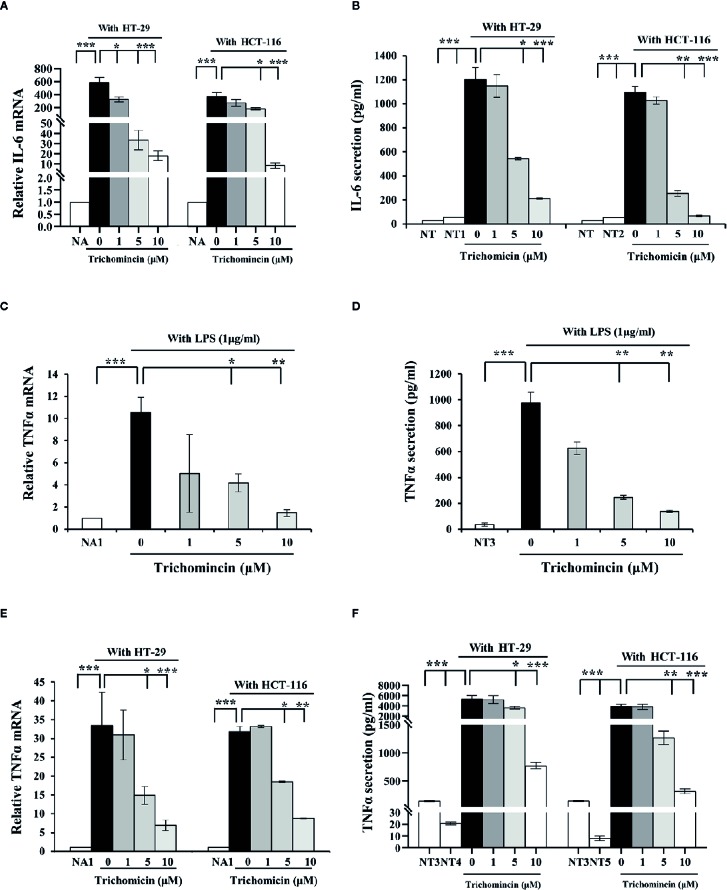
Trichomicin inhibits IL-6 and TNFα expression in macrophage. **(A)** Real-time PCR quantitation of IL-6 mRNA in macrophages stimulated with different CRC supernatants following treatment with 1, 5, and 10 μM Trichomicin. **(B)** Quantitation of IL-6 secretion in macrophages stimulated with different CRC supernatants following treatment with 1, 5, and 10 μM Trichomicin using ELISA. **(C, E)** Real-time PCR quantitation of TNFα mRNA in macrophages stimulated with LPS **(C)**, and different CRC supernatants **(E)** following treatment with 1, 5, and 10 μM Trichomicin. **(D, F)** Quantitation of TNFα secretion in macrophages stimulated with LPS **(D)** and different CRC supernatants **(F)** following treatment with 1, 5, and 10 μM Trichomicin. NA: Expression of IL-6 mRNA in untreated macrophages. NA1: Expression of TNFα mRNA in untreated macrophages. NT: Secretion of IL-6 in untreated macrophages cells. NT1: Levels of IL-6 in the culture media of HT-29 cells. NT2: Levels of IL-6 in the culture supernatant of HCT-116 cells. NT3: Secretion of TNFα in untreated macrophages cells. NT4: Levels of TNFα in the culture media of HT-29 cells. NT5: Levels of TNFα in the culture supernatant of HCT-116 cells. (n≥3) *P < 0.05, **P < 0.01, ***P < 0.001.

A previous study showed that Trichomicin reduced transcription and secretion of IL-6 in macrophages stimulated with LPS. The results showed that 10μM, Trichomicin reduced the levels of IL-6 mRNA by 92.22% and reduced secretion of Trichomicin by 82.96% in LPS-stimulated macrophages (Zhu et al., 2020). Our results showed that the expression of IL-6 mRNA in THP1 macrophages was enhanced by supplementation with culture supernatants from HT-29 and HCT-116 cells. Furthermore, Trichomicin inhibited the expression of IL-6 mRNA in macrophages treated with CRC supernatant in a dose-dependent manner. In macrophages stimulated with HT-29 supernatants, the expression of IL-6 mRNA was decreased to 44.51% (P < 0.05), 94.6% (P < 0.001), and 96.96% (P < 0.001), and with HCT-116 supernatants was decreased to 27.99%, 51.34% (P < 0.05), and 97.85% (P < 0.001) in response to 1, 5, and 10 μM Trichomicin, respectively (**Figure 2A**). Furthermore, the mRNA level of IL-6 in macrophages stimulated with CRC supernatants was 24-fold higher than that in cells treated with LPS. In addition, IL-6 secretion from macrophages showed a similar pattern as mRNA expression (**Figure 2B**).

The transcription and secretion levels of TNFα in macrophages were increased in response to LPS or culture supernatants from HT-29 and HCT-116. Furthermore, Transcription and secretion of TNFα in macrophages stimulated with CRC supernatants were also higher than those in macrophages treated with LPS. Trichomicin inhibited transcription and secretion of TNFα in macrophages in a dose-dependent manner (**Figures 2C–F**).

### Trichomicin Downregulate TNFα–Induced NF-κB Pathway Activation and Blocked Basal Stat3 Phosphorylation in Cancer Cells in an NF-κB-Independent Manner

**Figure 3:**
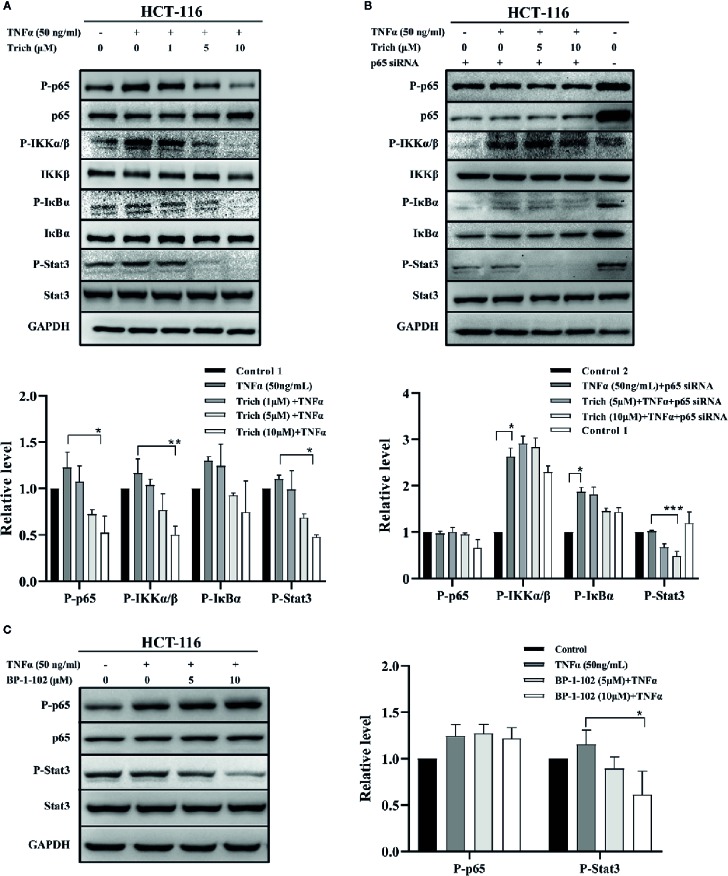
Trichomicin inhibited phosphorylation of components of the NF-κB signaling pathway. **(A)** Effect of Trichomicin on TNFα–induced phosphorylation of NF-κB p65, IKKα/β, IκBα and basal Stat3 phosphorylation in HCT-116 cells. **(B)** The effect of Trichomicin on the NF-κB and Stat3 signaling pathway in HCT-116 transfected with NF-κB p65 siRNA. **(C)** Immunoblotting analysis of TNFα–induced phosphorylation of NF-κB p65 and basal Stat3 phosphorylation in HCT-116 cells treated with BP-1-102. HCT-116 cells were preincubated with Trichomicin or BP-1-102 for 1 h prior to treatment with TNFα for 1 h. Whole cell lysates were prepared for western blotting. (n≥3) *P < 0.05, **P < 0.01, ***P < 0.001.

Since Trichomicin downregulated TNFα expression in tumor tissues and TAMs, we examined whether Trichomicin could inhibit TNFα–induced NF-κB activation in HCT-116 cells. The phosphorylation levels of NF-κB p65, IKKα/β, IκBα, and Stat3 were evaluated using western blot. Phosphorylation of NF-κB p65, IKKα/β, and IκBα were enhanced following TNFα stimulation, and were significantly inhibited by treatment with 5 and 10 μM Trichomicin (**Figure 3A**). Furthermore, 5 and 10 μM Trichomicin blocked basal Stat3 phosphorylation. To determine whether inhibition of basal Stat3 phosphorylation by trichomicin was caused by downregulation of the NF-κB pathway, HCT-116 cells were transfected with p65 siRNA and control siRNA for 72 h prior to exposure to TNFα and Trichomicin. Following transfection, treatment with TNFα did not induce NF-κB and IκBα activation, and Trichomicin did not inhibit phosphorylation of p65 and IκBα following NF-κB knockdown. However, Trichomicin was still able to block basal Stat3 phosphorylation (**Figure 3B**). This result showed that the inhibitory effect of Trichomicin on Stat3 phosphorylation was NF-κB-independent in CRC cell lines.

Parallel experiments were performed using the Stat3 inhibitor BP-1-102. As shown in **Figure 3C**, 5 and 10 μM BP-1-102 significantly suppressed basal Stat3 phosphorylation, but had no effect on TNFα–induced p65 phosphorylation. In contrast, a previous study showed that BP-1-102 decreased nuclear p65 phosphorylation in MDA-MB-231 cells (Zhang et al., 2012). This discrepancy may have been due to lower concentrations of BP-1-102 in our study. In addition, we noticed signs of cytotoxicity for HCT-116 cells when the concentration of BP-1-102 exceeded 10 μM.

### Trichomicin Inhibited Phosphorylation of the NF-κB Pathway and Stat3 in TAMs Stimulated With CRC Cell Lines

**Figure 4:**
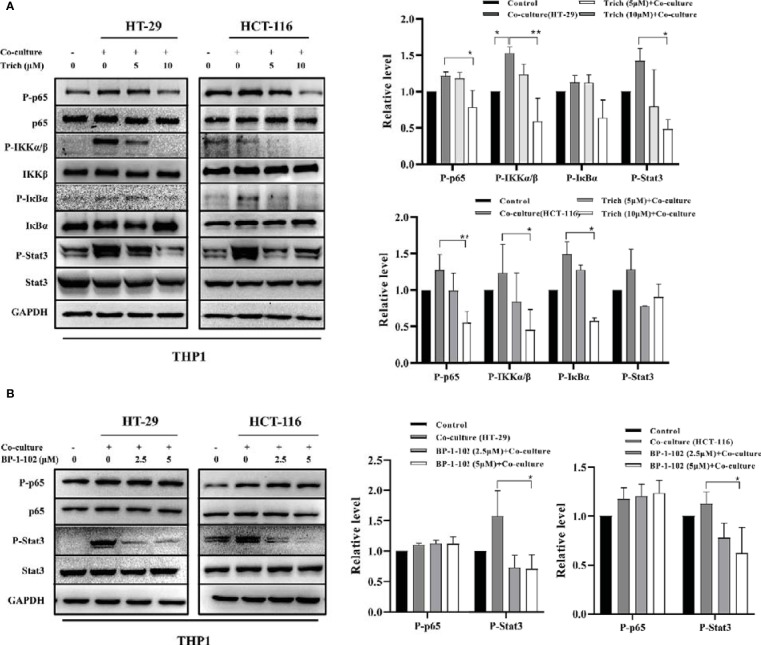
Effect of Trichomicin on the NF-κB pathway and Stat3 signal pathway in TAM. **(A)** The effect of Trichomicin on signal pathway in THP1 macrophage cells stimulated by supernatant of HT-29 and HCT-116. **(B)** The effect of BP-1-102 on signal pathway in THP1 macrophage cells treated with supernatant of HT-29 and HCT-116. (n≥3) *P < 0.05, **P < 0.01.

To further elucidate the role of Trichomicin in the CRC microenvironment, we evaluated signaling transduction pathways associated with TNFα and IL-6 expression in TAMs using immunoblotting. Phosphorylation of NF-κB p65, IKKα/β, IκBα and as Stat3 were analyzed following preincubation with Trichomicin for 1 h followed by stimulation with different CRC supernatants for 1 h. The results showed that phosphorylation of Stat3 was significantly enhanced, and phosphorylation of NF-κB p65, IKKα/β, and IκBα were increased in macrophages following stimulation with different CRC supernatants (**Figure 4A**). Treatment with 2.5 and 5 μM BP-1-102 inhibited Stat3 phosphorylation, but did not affect phosphorylation of NF-κB p65 in TAMs (**Figure 4B**).

### Trichomicin Suppressed the Phosphorylation of the NF-κB Pathway and Stat3 in CAFs Stimulated With CRC Cell Lines

**Figure 5:**
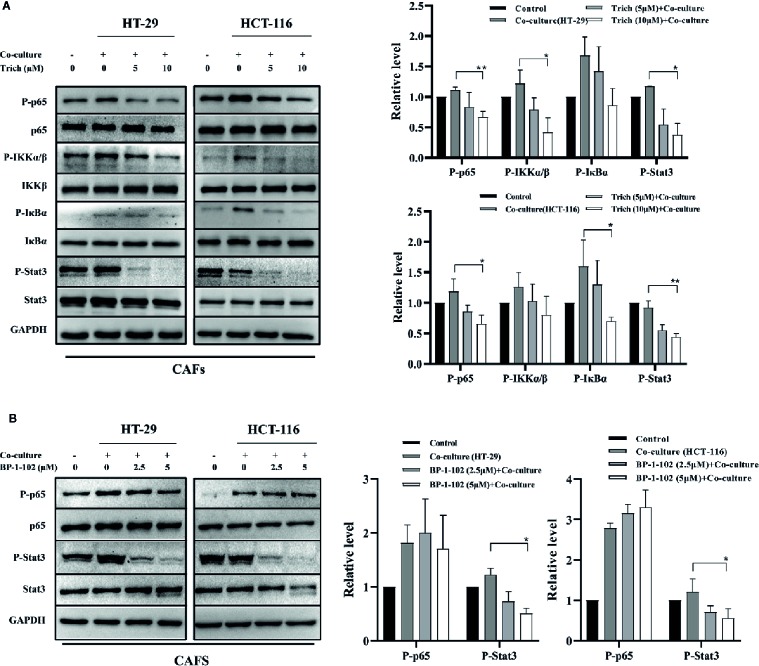
Effect of Trichomicin on NF-κB pathway and Stat3 signaling pathway expression in CAFs. **(A)** The effect of Trichomicin on signaling pathways in CAFs stimulated by supernatant of HT-29 and HCT-116. **(B)** The effect of BP-1-102 on signaling pathways in CAFs treated with supernatant of HT-29 and HCT-116. (n≥3) *P < 0.05, **P < 0.01.

Macrophages have historically been considered the main source of IL-6 and TNFα in CRC. New data, however, links IL-6 production to cancer associated fibroblasts (CAFs) in human colorectal cancer. Therefore, we further examined the effect of Trichomicin on CAFs isolated from specimens resected from human poorly-differentiated colorectal adenocarcinomas. Western blot results showed that the phosphorylation levels of p65, IKKα/β, IκBα, and Stat3 in CAFs treated with CRC supernatants were suppressed by Trichomicin (**Figure 5A**). Although 2.5 and 5μM of BP-1-102 could inhibit Stat3 phosphorylation, they had no effect on phosphorylation of NF-κB p65 in CAFs (**Figure 5B**).

### Trichomicin Inhibited PD-L1 Expression in CRC and Stromal Cells

**Figure 6:**
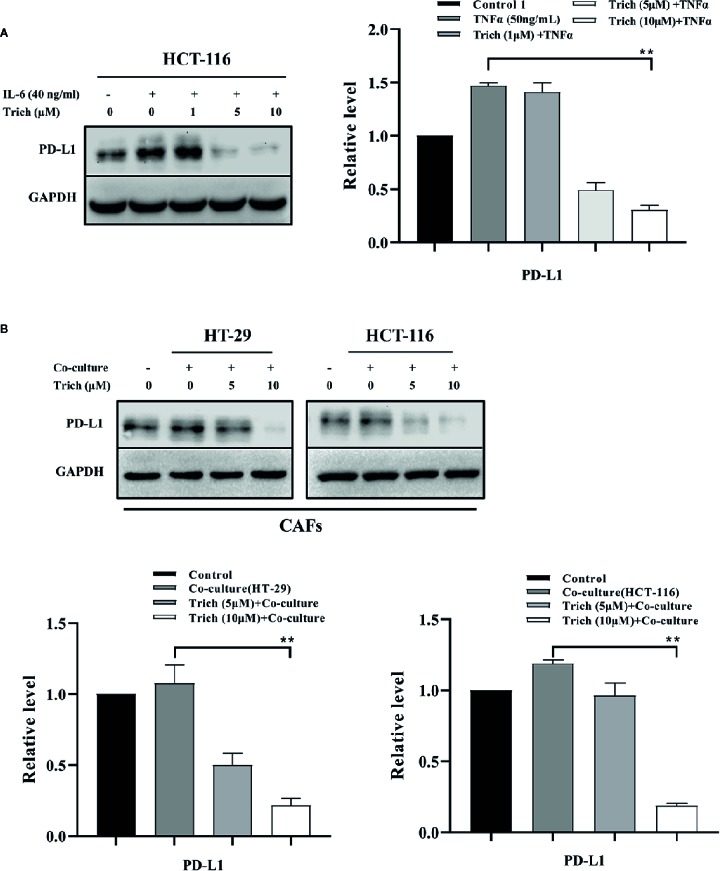
Trichomicin inhibited PD-L1 expression in CRC and stromal cells. **(A)** The effect of Trichomicin on PD-L1 in HCT-116 cells stimulated with IL-6. **(B)** The effect of Trichomicin on PD-L1 expression in CAFs treated with supernatant of HT-29 and HCT-116 cells. **P < 0.01.

Previous studies have shown that PD-L1 is expressed in lymphoma, and is transcriptionally regulated by Stat3 (Ansell et al., 2015; Atsaves et al., 2017). In our previous study, we showed that Trichomicin inhibited IL-6 expression and reduced IL-6-induced phosphorylation of Stat3 (Zhu et al., 2020). In this study, we found that Trichomicin inhibited phosphorylation of Stat3 in TAMs and CAFs. We then evaluated whether PD-L1 expression was regulated by Stat3 in CRC cell lines. The results showed that PD-L1 expression was enhanced by IL-6 stimulation in HCT-116 cells (**Figure 6A**), which was similar to IL-6-induced Stat3 phosphorylation in a previous study (Zhu et al., 2020). Furthermore, Trichomicin significantly inhibited PD-L1 expression in HCT-116 and CAF (**Figure 6B**) in a dose-dependent manner. In particular, 10 μM Trichomicin reduced PD-L1 expression further than basal levels. Although PD-L1 expression was not detected in HT-29 and macrophages, Trichomicin may have inhibited CRC *in vivo* by targeting the IL6-Stat3-PD-L1 pathway.

## Discussion

Our previous study showed that Trichomicin, a natural compound derived from *Trichoderma hazianum*, exerted potent anti-inflammatory and anticancer activity *in vitro* (Qi et al., 2011), and induced apoptosis in cancer cells through inhibition of IL-6/Stat3 signaling (Zhu et al., 2020). In this study, we evaluated the anticancer activity of Trichomicin *in vivo,* and found that it decreased the expression of IL-6 and TNFα in tumor tissues. The antitumor activity of Trichomicin was significantly stronger than that of siltuximab (Angevin et al., 2014) (an anti-IL-6 monoclonal antibody) and infliximab (Flores et al., 2012) (an anti-TNFα monoclonal antibody). Trichomicin also showed good oral absorption and rapid uptake. Furthermore, Trichomicin exerted antitumor effects by targeting Stat3, NF-κB, and PD-L1 in tumor and stromal cells (**Graphical Abstract**). Trichomicin may be a strategy for treatment of colorectal cancer through modulation of the tumor microenvironment.

Interleukin-6 and TNFα are important mediators that link inflammation and cancer (Yao et al., 2014; Marelli et al., 2017). In CRC, IL-6 and TNFα participate in most steps of cancer progression, including tumor initiation, proliferation, migration, and angiogenesis (Flores et al., 2012; Wang et al., 2013; Wang and Sun, 2014; West et al., 2015). A previous study showed that production of IL-6 was increased in tumor tissue and serum of patients with CRC. High levels of IL-6 were associated with shorter survival time than that in patients with low IL-6 expressions (Wu et al., 1996). In addition, elevated serum levels of TNFα were associated with cancer stage and progression, and was linked to poor prognosis (Stanilov et al., 2014). In our study, Trichomicin reduced the levels of IL-6 and TNFα in colon cancer tumor tissues, which indicated that it had potential as an adjuvant treatment for colon cancer. Tumor-associated macrophages (TAMs) are a major source of IL-6 and TNFα in CRC (Schupp et al., 2019). Furthermore, IL-6 and TNFα are highly expressed in the malignant tumor microenvironment, which can promote tumor invasion, distant tumor metastasis, angiogenesis, and tumor resistance. To further evaluate crosstalk between Trichomicin and the tumor microenvironment *in vitro*, we investigated the ability of Trichomicin to reduce the expression and secretion of IL-6 and TNFα in macrophage-like differentiated THP1 cells. Furthermore, we found that the expression and secretion of IL-6 and TNFα were higher in macrophages stimulated with CRC supernatants than in cells treated with LPS, and this effect occurred in a dose-dependent manner. Treatment with 10μM Trichomicin, a non-cytotoxic concentration (**Figure S3**), inhibited transcription and secretion of IL-6 and TNFα by more than 90% (**Figure 2**).

The IL-6 and TNFα signaling pathways have been well characterized. Interleukin-6 induces homodimerization of the signal transducing glycoprotein gp130 following binding to the IL-6 receptor (IL-6R), which results in activation of JAK/STAT signal transduction pathway (Rose-John, 2012; Liu et al., 2018). In addition, TNFα activates the IκB kinase (IKK) complex, which phosphorylates IκB. Phosphorylated IκB is recognized by the ubiquitin ligase machinery, resulting in ubiquitination and subsequent degradation, which allows translocation of free NF-κB dimer to the nucleus (Hayden and Ghosh, 2004). Cytokine-mediated JAK/Stat3 pathway activation has been shown to promote excessive proliferation and invasion of colon cancer cells (Gordziel et al., 2013). The NF-κB signaling pathway plays an important role in regulation of inflammation and apoptosis. It is constitutively activated in various human tumors and is associated with tumor development (Dolcet et al., 2005). Conditionally inactivated NF-κB in myeloid cells significantly reduced the expression of many inflammation-related genes, such as IL-1β, TNFα, human macrophage inflammatory protein, and cyclooxgenase 2, and tumor size significantly decreased in mice (Pikarsky et al., 2004). Therefore, we examined the effect of Trichomicin on IL-6-induced phosphorylation of Stat3 (Zhu et al., 2020) and TNFα-induced activation of the NF-κB pathway in CRC cells. Trichomicin inhibited TNFα-induced NF-kB phosphorylation and induced activation of Stat3-related signaling pathways in a dose-dependent manner in colon cancer cells. Expression of cytokines in response to NF-κB results in activation of Stat3, which plays a key role in controlling communication between cancer cells and the surrounding microenvironment (Grivennikov and Karin, 2010). Our results showed that Trichomicin blocked basal Stat3 phosphorylation in an NF-κB-independent manner.

Cancer-stromal interactions support and maintain tumor growth (Cammarota et al., 2010). Tumor-associated macrophages are a major source of IL-6 and TNFα in CRC (West et al., 2015; van den Bosch et al., 2017; Zhu and Cao, 2017; Schupp et al., 2019). Recent studies have shown that cancer stromal fibroblasts (CAFs) in CRC are an important source of IL-6, which is critical for tumor angiogenesis (Nagasaki et al., 2014; Huynh et al., 2016). To further characterize targets of Trichomicin in the tumor microenvironment, we investigated the effects of Trichomicin on the Stat3 and NF-κB pathways in CAFs stimulated with CRC supernatants. Our results showed that Trichomicin inhibited CRC conditioned media-induced phosphorylation of Stat3 and NF-κB in CAFs. Although BP-1-102 suppressed Stat3 phosphorylation, it had no effect on phosphorylation of NF-κB p65 in stromal cells. These findings indicated that Trichomicin exerted pleiotropic activity in the tumor inflammatory microenvironment, and may have promise as a treatment strategy for colorectal cancer through targeting of tumor-associated stromal cells.

The combination of chemotherapy and anti-angiogenic therapy is an important strategy for treatment of metastatic colorectal cancer (mCRC). However, CRC eventually becomes resistant to all available therapies (Amin and Lockhart, 2015). Recently, anti-PD1 drugs have been used to treat metastatic colorectal cancer in patients with high levels of microsatellite instability (MSI-H) or mismatch-repair-deficiency (dMMR), which generated enthusiasm for immunotherapy. Similarly, anti-PDL1 blockers are being studied as therapeutic agents for refractory metastatic solid tumors, including CRC (Boland and Ma, 2017). However, CRC is a poor candidate for immunotherapy because MSI only accounts for 15% of CRC cases, and the frequency of MSI-H is only 5.9% (Van der Jeught et al., 2018). In addition to regulation of the TME, Trichomicin reduced the expression of PD-L1 in HCT-116 and CAFs to below basal levels, which indicated that Trichomicin has potential as a PD-L1 targeted drug. Trichomicin, derived from a microorganism (Patent No: ZL 200510082974.X), exhibits low toxicity and good oral properties, and is a promising candidate for CRC treatment either alone or in combination with monoclonal antibodies.

## Conclusion

Trichomicin, a promising compound with antitumor-activity, was validated for its antitumor effects in colon tumor-bearing mice. Trichomicin exerted antitumor effects through the TNFα-NF-κB and IL6-Stat3 pathways, and the inhibitory effect of Trichomicin on Stat3 was NF-κB-independent. Furthermore, Trichomicin regulated the TME, and inhibited the expression of the immune check point protein PD-L1 in cancer and stromal cells. Demonstration of oral bioavailability and anticancer activity of Trichomicin showed that this molecule may be an effective treatment for colorectal cancer.

## Data Availability Statement

All datasets generated for this study are included in the article/**Supplemental Material**.

## Ethics Statement

The animal study was reviewed and approved by Peking Union Medical College (PUMC) Pharmaceutical Institutional Animal Care and Use Committee.

## Author Contributions

LB, XZ, and XQ conceived of the study and designed the experiments. XZ, XQ, WL, ZZ, and XT performed the experiments. PW, HW, and LS performed the statistical analyses. XZ, JG, XQ, and LB wrote the manuscript. All authors read and approved the final manuscript.

## Funding

This research was supported by grants from National Key Research and Development Program of China (2018YFA0902000), the Special Fund of Chinese Central Government for Basic Scientific Research Operations in Commonweal Research Institutes (2016ZX350056), and CAMS Innovation Fund for Medical Sciences (2017-I2M-1-012).

## Conflict of Interest

The authors declare that the research was conducted in the absence of any commercial or financial relationships that could be construed as a potential conflict of interest.

## Abbreviations

TME, Tumor microenvironment; TAMs, Tumor associated macrophages; CRC, Colorectal cancer; NF-κB, Nuclear factor κB; Stat3, Signal transducer and activator of transcription 3; IKK, IκB kinase; GAPDH, Glyceraldehyde-3-phosphate dehydrogenase; PD-1, Programmed cell death protein 1; PD-L1, Programmed cell death Ligand 1; IL-6, Interleukin-6; TNFα, Tumor Necrosis Factor-α; LPS, Lipopolysaccharide; siRNA, Small interfering RNA; PMA, Phorbol 12-myristate 13-acetate; STR, Short tandem repeat; CAFs, Colon carcinoma associated fibroblasts; FBS, Fetal bovine serum; PBS, Phosphate buffer saline; DMSO, Dimethyl sulfoxide; CMC, Carboxymethylcellulose; ELISA, Enzyme Linked Immunosorbent Assay; PCR, Polymerase Chain Reaction.

## References

[B1] Adams J. L. Smothers J. Srinivasan R. Hoos A. (2015). Big opportunities for small molecules in immuno-oncology. Nat. Rev. Drug Discovery 14 (9), 603–622. 10.1038/nrd4596 26228631

[B2] Amin M. Lockhart A. C. (2015). The potential role of immunotherapy to treat colorectal cancer. Expert Opin. Invest. Drugs 24 (3), 329–344. 10.1517/13543784.2015.985376 25519074

[B3] Angevin E. Tabernero J. Elez E. Cohen S. J. Bahleda R. van Laethem J. L. (2014). A phase I/II, multiple-dose, dose-escalation study of siltuximab, an anti-interleukin-6 monoclonal antibody, in patients with advanced solid tumors. Clin. Cancer Res. 20 (8), 2192–2204. 10.1158/1078-0432.CCR-13-2200 24563479

[B4] Ansell S. M. Lesokhin A. M. Borrello I. Halwani A. Scott E. C. Gutierrez M. (2015). PD-1 blockade with nivolumab in relapsed or refractory Hodgkin's lymphoma. N. Engl. J. Med. 372 (4), 311–319. 10.1056/NEJMoa1411087 25482239 PMC4348009

[B5] Atsaves V. Tsesmetzis N. Chioureas D. Kis L. Leventaki V. Drakos E. (2017). PD-L1 is commonly expressed and transcriptionally regulated by STAT3 and MYC in ALK-negative anaplastic large-cell lymphoma. Leukemia 31 (7), 1633–1637. 10.1038/leu.2017.103 28344319

[B6] Balkwill F. (2009). Tumour necrosis factor and cancer. Nat. Rev. Cancer 9 (5), 361–371. 10.1038/nrc2628 19343034

[B7] Boland P. M. Ma W. W. (2017). Immunotherapy for Colorectal Cancer. Cancers (Basel) 17 (6), 709–721. 10.3390/cancers9050050 PMC5447960 28492495

[B8] Bollrath J. Phesse T. J. von Burstin V. A. Putoczki T. Bennecke M. Bateman T. (2009). gp130-mediated Stat3 activation in enterocytes regulates cell survival and cell-cycle progression during colitis-associated tumorigenesis. Cancer Cell 15 (2), 91–102. 10.1016/j.ccr.2009.01.002 19185844

[B9] Cammarota R. Bertolini V. Pennesi G. Bucci E. O. Gottardi O. Garlanda C. (2010). The tumor microenvironment of colorectal cancer: stromal TLR-4 expression as a potential prognostic marker. J. Transl. Med. 8, 112. 10.1186/1479-5876-8-112 21059221 PMC2997091

[B10] Coskun O. Oztopuz O. Ozkan O. F. (2017). Determination of IL-6, TNF-alpha and VEGF levels in the serums of patients with colorectal cancer. Cell Mol. Biol. (Noisy-le-grand) 63 (5), 97–101. 10.14715/cmb/2017.63.5.18 28719352

[B11] Crusz S. M. Balkwill F. R. (2015). Inflammation and cancer: advances and new agents. Nat. Rev. Clin. Oncol. 12 (10), 584–596. 10.1038/nrclinonc.2015.105 26122183

[B12] De Simone V. Franze E. Ronchetti G. Colantoni A. Fantini M. C. Di Fusco D. (2015). Th17-type cytokines, IL-6 and TNF-alpha synergistically activate STAT3 and NF-kB to promote colorectal cancer cell growth. Oncogene 34 (27), 3493–3503. 10.1038/onc.2014.286 25174402 PMC4493653

[B13] Dekker E. Tanis P. J. Vleugels J. L. A. Kasi P. M. Wallace M. B. (2019). Colorectal cancer. Lancet 394 (10207), 1467–1480. 10.1016/s0140-6736(19)32319-0 31631858

[B14] Dolcet X. Llobet D. Pallares J. Matias-Guiu X. (2005). NF-kB in development and progression of human cancer. Virchows Arch. 446 (5), 475–482. 10.1007/s00428-005-1264-9 15856292

[B16] Eyob H. Ekiz H. A. Welm A. L. (2013). RON promotes the metastatic spread of breast carcinomas by subverting antitumor immune responses. Oncoimmunology 2 (9), e25670. 10.4161/onci.25670 24327933 PMC3850023

[B17] Flores M. B. S. Rocha G. Z. Damas-Souza D. M. Osorio-Costa F. Dias M. M. Ropelle E. R. (2012). RETRACTED: Obesity-induced increase in tumor necrosis factor-alpha leads to development of colon cancer in mice. Gastroenterology 143 (3), 741–753 e744. 10.1053/j.gastro.2012.05.045 22677195

[B18] Ganesh K. Stadler Z. K. Cercek A. Mendelsohn R. B. Shia J. Segal N. H. (2019). Immunotherapy in colorectal cancer: rationale, challenges and potential. Nat. Rev. Gastroenterol. Hepatol. 16 (6), 361–375. 10.1038/s41575-019-0126-x 30886395 PMC7295073

[B19] Gordziel C. Bratsch J. Moriggl R. Knösel T. Friedrich K. (2013). Both STAT1 and STAT3 are favourable prognostic determinants in colorectal carcinoma. Br. J. Cancer 109 (1), 138–146. 10.1038/bjc.2013.274 23756862 PMC3708576

[B21] Grivennikov S. I. Karin M. (2010). Dangerous liaisons: STAT3 and NF-kappaB collaboration and crosstalk in cancer. Cytokine Growth Factor Rev. 21 (1), 11–19. 10.1016/j.cytogfr.2009.11.005 20018552 PMC2834864

[B20] Grivennikov S. Karin E. Terzic J. Mucida D. Yu G. Y. Vallabhapurapu S. (2009). IL-6 and Stat3 are required for survival of intestinal epithelial cells and development of colitis-associated cancer. Cancer Cell 15 (2), 103–113. 10.1016/j.ccr.2009.01.001 19185845 PMC2667107

[B23] Hayden M. S. Ghosh S. (2004). Signaling to NF-kappaB. Genes Dev. 18 (18), 2195–2224. 10.1101/gad.1228704 15371334

[B24] Huynh P. T. Beswick E. J. Coronado Y. A. Johnson P. O'Connell M. R. Watts T. (2016). CD90(+) stromal cells are the major source of IL-6, which supports cancer stem-like cells and inflammation in colorectal cancer. Int. J. Cancer 138 (8), 1971–1981. 10.1002/ijc.29939 26595254 PMC4865268

[B25] Koumenis C. Hammond E. Giaccia A. (2014). Tumor microenvironment and cellular stress: signaling, metabolism, imaging, and therapeutic targets. Preface. Adv. Exp. Med. Biol. 772, v–viii. 10.1007/978-1-4614-5915-6 24765667

[B26] Liu X. Su X. Xu S. Wang H. Han D. Li J. (2018). MicroRNA in vivo precipitation identifies miR-151-3p as a computational unpredictable miRNA to target Stat3 and inhibits innate IL-6 production. Cell Mol. Immunol. 15 (2), 99–110. 10.1038/cmi.2017.82 28890541 PMC5811685

[B27] Marelli G. Sica A. Vannucci L. Allavena P. (2017). Inflammation as target in cancer therapy. Curr. Opin. Pharmacol. 35, 57–65. 10.1016/j.coph.2017.05.007 28618326

[B28] Miranda D. O. Anatriello E. Azevedo L. R. Cordeiro J. F. C. Peria F. M. Floria-Santos M. (2018). Elevated serum levels of proinflammatory cytokines potentially correlate with depression and anxiety in colorectal cancer patients in different stages of the antitumor therapy. Cytokine 104, 72–77. 10.1016/j.cyto.2017.09.030 28969939

[B29] Nagasaki T. Hara M. Nakanishi H. Takahashi H. Sato M. Takeyama H. (2014). Interleukin-6 released by colon cancer-associated fibroblasts is critical for tumour angiogenesis: anti-interleukin-6 receptor antibody suppressed angiogenesis and inhibited tumour-stroma interaction. Br. J. Cancer 110 (2), 469–478. 10.1038/bjc.2013.748 24346288 PMC3899773

[B30] Pedrosa L. Esposito F. Thomson T. M. Maurel J. (2019). The Tumor Microenvironment in Colorectal Cancer Therapy. Cancers (Basel) 11 (8), E1172. 10.3390/cancers11081172 31416205 PMC6721633

[B31] Pikarsky E. Porat R. M. Stein I. Abramovitch R. Amit S. Kasem S. (2004). NF-kappaB functions as a tumour promoter in inflammation-associated cancer. Nature 431 (7007), 461–466. 10.1038/nature02924 15329734

[B32] Popivanova B. K. Kitamura K. Wu Y. Kondo T. Kagaya T. Kaneko S. (2008). Blocking TNF-alpha in mice reduces colorectal carcinogenesis associated with chronic colitis. J. Clin. Invest. 118 (2), 560–570. 10.1172/JCI32453 18219394 PMC2213370

[B33] Qi X. Q. Zhu F. C. Yang Z. Guo L. H. Yuan L. (2011). Study of a novel compound 2460A with activities produced by fungus. Yao xue xue bao = Acta Pharm. Sin. 46 (2), 165–169. 21542287

[B34] Rose-John S. (2012). IL-6 trans-signaling via the soluble IL-6 receptor: importance for the pro-inflammatory activities of IL-6. Int. J. Biol. Sci. 8 (9), 1237–1247. 10.7150/ijbs.4989 23136552 PMC3491447

[B35] Schupp J. Krebs F. K. Zimmer N. Trzeciak E. Schuppan D. Tuettenberg A. (2019). Targeting myeloid cells in the tumor sustaining microenvironment. Cell Immunol. 343, 103713. 10.1016/j.cellimm.2017.10.013 29129292

[B36] Stanilov N. Miteva L. Dobreva Z. Stanilova S. (2014). Colorectal cancer severity and survival in correlation with tumor necrosis factor-alpha. Biotechnol. Biotechnol. Equip. 28 (5), 911–917. 10.1080/13102818.2014.965047 26019577 PMC4433886

[B37] van den Bosch T. P. P. Kannegieter N. M. Hesselink D. A. Baan C. C. Rowshani A. T. (2017). Targeting the Monocyte–Macrophage Lineage in Solid Organ Transplantation. Front. Immunol. 8, 153. 10.3389/fimmu.2017.00153 28261211 PMC5312419

[B15] Van der Jeught K. Xu H. C. Li Y. J. Lu X. B. Ji G. (2018). Drug resistance and new therapies in colorectal cancer. World J. Gastroenterol. 24 (34), 26–40. 10.3748/wjg.v24.i34.3834 PMC6141340 30228778

[B39] Wang S. W. Sun Y. M. (2014). The IL-6/JAK/STAT3 pathway: potential therapeutic strategies in treating colorectal cancer (Review). Int. J. Oncol. 44 (4), 1032–1040. 10.3892/ijo.2014.2259 24430672

[B38] Wang H. Wang H. S. Zhou B. H. Li C. L. Zhang F. Wang X. F. (2013). Epithelial-mesenchymal transition (EMT) induced by TNF-alpha requires AKT/GSK-3beta-mediated stabilization of snail in colorectal cancer. PloS One 8 (2), e56664. 10.1371/journal.pone.0056664 23431386 PMC3576347

[B40] West N. R. McCuaig S. Franchini F. Powrie F. (2015). Emerging cytokine networks in colorectal cancer. Nat. Rev. Immunol. 15 (10), 615–629. 10.1038/nri3896 26358393

[B41] Wu C. W. Wang S. R. Chao M. F. Wu T. C. Lui W. Y. P'Eng F. K. (1996). Serum interleukin-6 levels reflect disease status of gastric cancer. Am. J. Gastroenterol. 91, 7, 1417–1422. 8678006

[B42] Yao X. Huang J. Zhong H. Shen N. Faggioni R. Fung M. (2014). Targeting interleukin-6 in inflammatory autoimmune diseases and cancers. Pharmacol. Ther. 141 (2), 125–139. 10.1016/j.pharmthera.2013.09.004 24076269

[B43] Zhang X. Yue P. Page B. D. Li T. Zhao W. Namanja A. T. (2012). Orally bioavailable small-molecule inhibitor of transcription factor Stat3 regresses human breast and lung cancer xenografts. Proc. Natl. Acad. Sci. U. S. A 109 (24), 9623–9628. 10.1073/pnas.1121606109 22623533 PMC3386073

[B44] Zhang Y. Wang L. Bai L. Jiang R. Guo L. Wu J. (2016). Effect of ebosin on modulating interleukin-1beta-induced inflammatory responses in rat fibroblast-like synoviocytes. Cell Mol. Immunol. 13 (5), 584–592. 10.1038/cmi.2015.36 25938977 PMC5037274

[B46] Zhu H. Cao X. (2017). NLR members in inflammation-associated carcinogenesis. Cell Mol. Immunol. 14 (5), 403–405. 10.1038/cmi.2017.14 28366939 PMC5423091

[B45] Zhu F. Zhao X. Li J. Guo L. Bai L. Qi X. (2020). A new compound Trichomicin exerts antitumor activity through STAT3 signaling inhibition. Biomed. Pharmacother. 121, 109608. 10.1016/j.biopha.2019.109608 31707338

[B47] Zitvogel L. Pietrocola F. Kroemer G. (2017). Nutrition, inflammation and cancer. Nat. Immunol. 18 (8), 843–850. 10.1038/ni.3754 28722707

