# Supplementary material for: Trichomicin Suppresses Colorectal Cancer via Comprehensive Regulation of IL-6 and TNFα in Tumor Cells, TAMs, and CAFs
Source: Front Pharmacol. 2020 Apr 3;11:386. doi: 10.3389/fphar.2020.00386 (PMC7146085; doi:10.3389/fphar.2020.00386)

**Trichomicin suppresses colorectal cancer via** **comprehensive regulation of IL-6 and TNFα in tumor cells, TAMs and CAFs**

Xi Zhao^a,1^, Xiaoqiang Qi^a, b, 1^, Wenrui Lian^a, c^, Xin Tong^a, c^, Hua Wang^c^, Liya Su^c^, Ping Wei^d^, Zhuochen Zhuang^a^, Jianhua Gong^a*^, Liping Bai^a*^

^a^ NHC Key Laboratory of Biotechnology of Antibiotics, Institute of Medicinal Biotechnology, Chinese Academy of Medical Sciences & Peking Union Medical College, 1 Tian Tan Xi Li, Beijing 100050, China

^b^ Department of Surgery and Ellis Fischel Cancer Center, University of Missouri-Columbia, Columbia, MO, 65212, United States

^c^ The Affiliated Hospital of Inner Mongolia Medical University, Hohhot 010050, China

^d^ Department of Medical Immunology, Basic Medical College, Shandong First Medical University & Shandong Academy of Medical Sciences, Taian 271000, China

^1^X. Zhao and X. Qi contributed equally to this work.

Corresponding author：Prof. Jianhua Gong and Prof. Liping Bai

Prof. Jianhua Gong; Phone: +861063024341; E-mail: [ann_gong@hotmail.com](mailto:ann_gong@hotmail.com)

Prof. Liping Bai; Phone: +861063013336; E-mail: [lipingbai1973@163.com](mailto:lipingbai1973@163.com)

Correspondence should be addressed to: Institute of Medicinal Biotechnology, Chinese Academy of Medical Sciences & Peking Union Medical College, 100050，Tian Tan Xi Li No.1，Beijing, China

Supplemental data

**Supplemental Materials and Methods**

**Transport and Uptake of** **Trichomincin**

For transport of Trichomincin, Caco-2 cells were seeded at 1×10^5^ cells per well in inserts containing permeable polycarbonate membrane (0.4μm pore size, 1.1 cm^2^ growth area) in 12-well Transwells^®^ plates (Corning, NY, USA) and incubated at 37 °C in 5% CO_2_ with fresh media replaced every 2 days. The quality of the monolayers grown on the permeable membrane was assessed by the transepithelial electrical resistances using Millicell-ERS (Millipore, MA, USA) weekly. Caco-2 cell monolayers cultured for 21 days (Resistances more than 600 Ω/cm^2^) were used for transport experiments according to previously described procedures[1]. *P*_app_ values (cm/sec) were calculated from the linear plot of drug transported to the acceptor side vs. time using the equation:

dQ/dt is the appearance rate of drug on the receiver side, C_0_ is the initial drug concentration, and A is the membrane surface area (1.1 cm^2^).

**Preliminary evaluation of** **acute preclinical toxicity and** **pharmacokinetics**

The evaluation of acute preclinical toxicity of Trichomincin was performed using Kunming mice. Mice were subjected to a single dose of 1.2 g/kg of Trichomicin in 0.5% Tween 80 via lavage administration. After administration, the animals were observed carefully for any gross effects or mortality. Weights, symptoms and deaths were recorded. After 18 days, all survived mice were sacrificed for autopsy to determine whether all organs were normal.

The evaluation of pharmacokinetics of Trichomincin was performed in female Wistar rats (body weight 200g). Rats were subjected to a single dose of 200 mg/kg of Trichomicin in 0.5% Tween 80 via lavage administration. Blood samples (0.5 mL) were collected into lithium-heparin plasma separator tubes at 0.5, 1, 2, 4, 8, 12, 24h post-administration. The collected samples were centrifuged at 10, 000g for 10 min, and the plasma was obtained for HPLC analysis.

**Cell viability assay**

Trichomincin cytotoxicity was analyzed by carrying out triplicated MTT assays. Cells were seeded in 96-well plates (1×10^4^ cells/well) and then exposed to Trichomincin as indicated. After 48 h, cells were incubated with MTT solution (20 μL, 5 mg/mL) for 3-4h. Subsequently, the medium was removed and 150 μl of DMSO was added to each well to dissolve the formazan crystals. The absorbance (A) at 570 nm was determined using a microplate reader (Thermo Fisher Scientific, MA, USA). The relative cell viability (%) compared with the control was calculated from the following formula: cell viability (%) = [(A_sample_–A_blank_)/(A_control_–A_blank_)] × 100%.

**Supplemental Results:**

**Trichomincin’s capability assay in cell absorption**

A monolayer of Caco-2 cells which has been a standard model to predict drug permeability [2] was used to characterize Trichomicin in cell absorption. Metoprolol and Atenolol were also included in this assay as control compounds (Supplementary Fig. S1 a and b). As expected, the Caco-2 monolayers exhibited higher permeability for Metoprolol (*P*_app_=1.44x10^-5^) compared with Atenolol (*P*_app_=2.95x10^-7^), indicating the integrity of the monolayers, meanwhile the permeability of Trichomicin (*P*_app_=8.2x10^-6^) was slightly lower than metoprolol but higher than Atenolol in the AP−BL direction. It showed that Trichomicin has the permeability and possibility to serve as an oral drug.

**Trichomicin’s plasma stability assay**

The preliminary pharmacokinetic study of Trichomicin was performed in rats. Trichomicin was detectable in plasma 30 min post oral administration at 200 mg/kg, and it reached the maximum plasma concentration (C_max_) of 4.8 μM at 4h post administration (Supplementary Figure S2). The elimination half-life (T_1/2_) was 4.85 ± 0.4 h.

**Trichomicin is not** **cytotoxic to** **macrophage and CRC cells *in vitro***

To understand the potential mechanisms underlying the *in vivo* antitumor effect of Trichomicin, we performed cell viability assays using macrophage and CRC cell lines. As the C_max_ of Trichomicin was 4.8 μM *in vivo*, the maximum concentration of 10 μM was used for cell viability and all subsequent cell assays. Three cell lines (macrophage-like differentiated THP-1 cells, HT-29, HCT-116) were cultured with varying concentrations of Trichomicin (0, 1, 2, 3, 4, 5, 10 μM) for 48 h, and cell viability was determined by the MTT assay. Supplementary Fig. S3 showed that Trichomicin did not inhibit the proliferation of these cells even at maximum concentration.

**Supplemental Figures:**


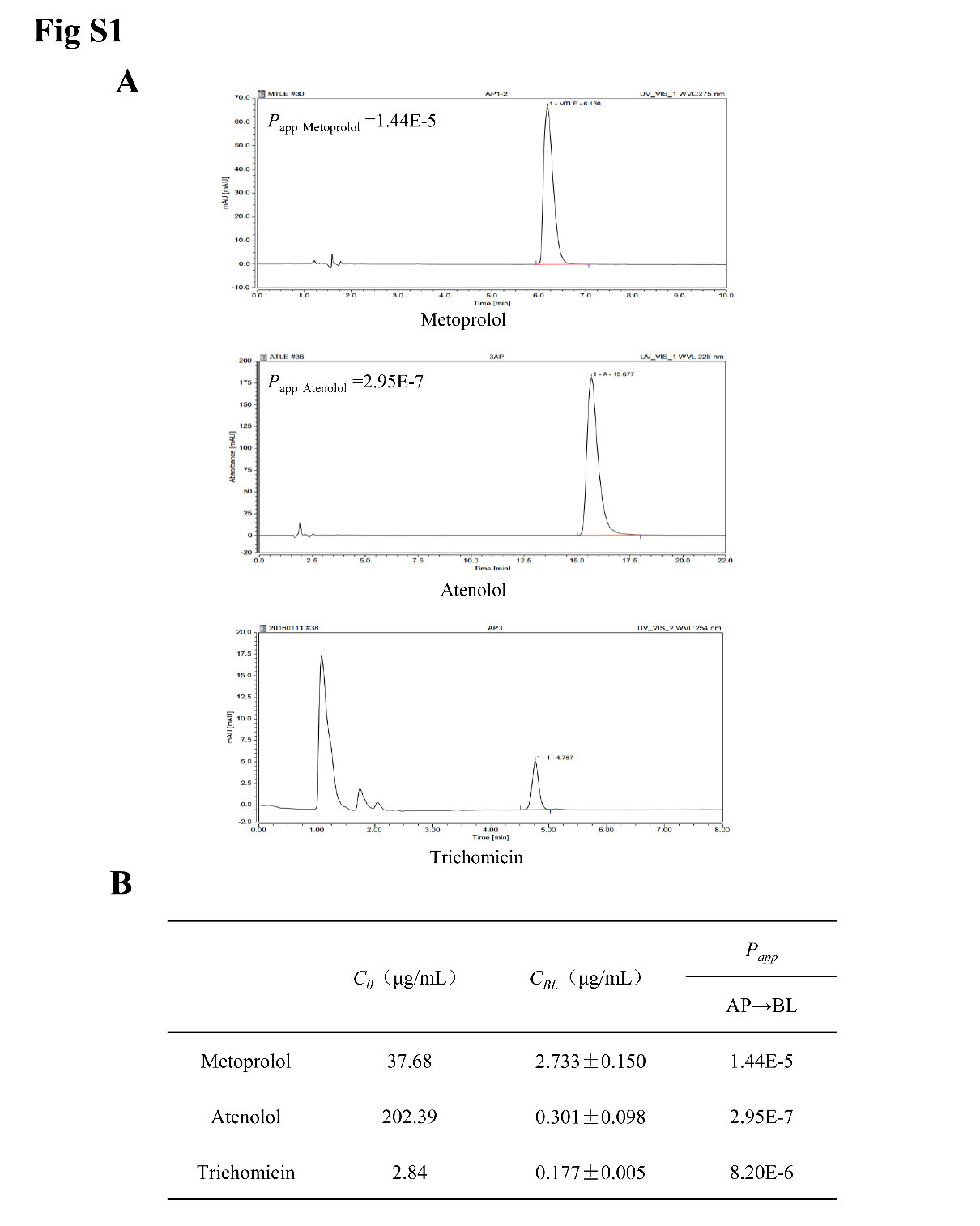


**Figure S1**

Transport analysis of Trichomincin. (a) The chromatogram of Metoprolol, Atenolol and Trichomicin transport across Caco-2 cells monolayers by HPLC. (b)


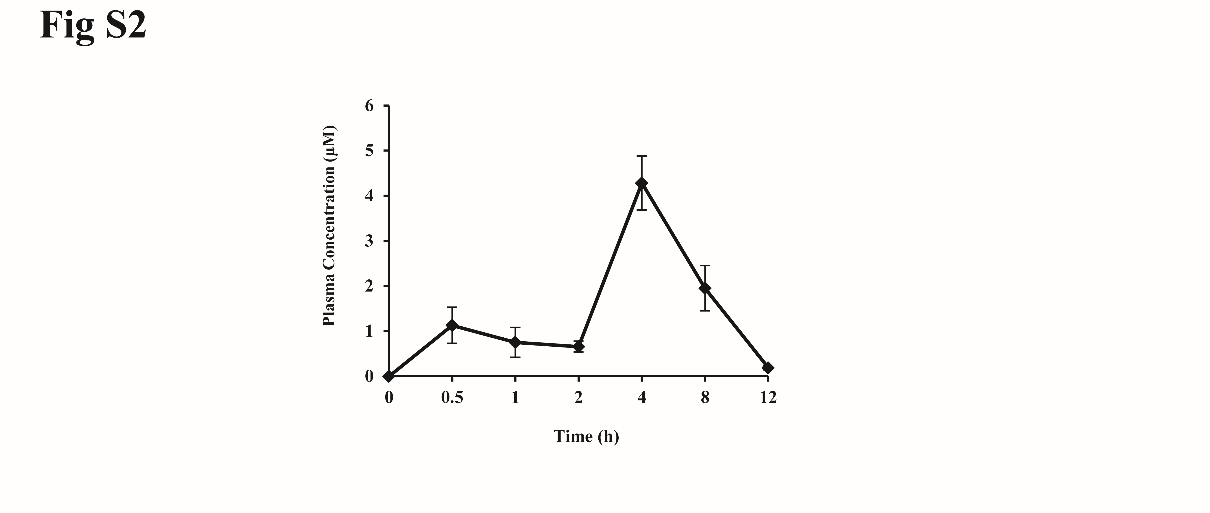


**Figure S2**

Mean plasma concentration-time profile of Trichomicin after oral administration.


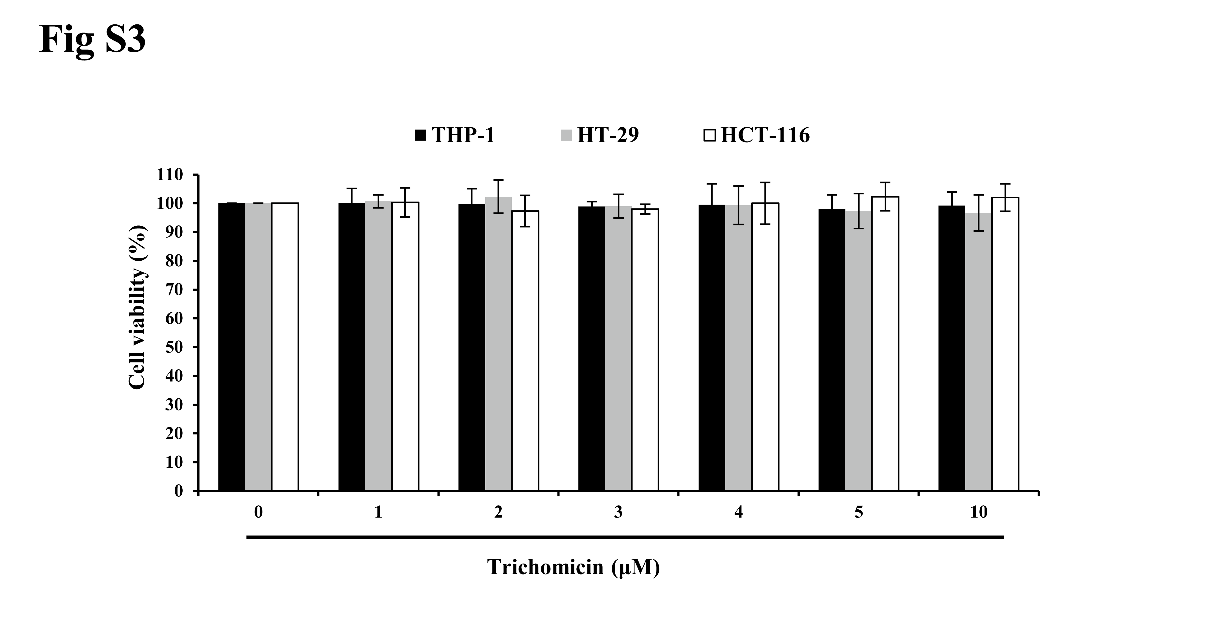


**Figure S3**

Effects of Trichomicin on the cell viability of THP-1, HT-29 and HCT-116 cells. Different cell lines were incubated with 0, 1, 2, 3, 4, 5, 10μM Trichomicin for 48h.

[1] Y. Yu, M. Huo, Y. Fu, W. Xu, H. Cai, L. Yao, Q. Chen, Y. Mu, J. Zhou, T. Yin, N-Deoxycholic acid-N,O-hydroxyethyl Chitosan with a Sulfhydryl Modification To Enhance the Oral Absorptive Efficiency of Paclitaxel, Mol Pharm 14(12) (2017) 4539-4550.

[2] F. Araujo, B. Sarmento, Towards the characterization of an in vitro triple co-culture intestine cell model for permeability studies, Int J Pharm 458(1) (2013) 128-34.

**Supplemental** **Western Blot Uncropped Original Figures**


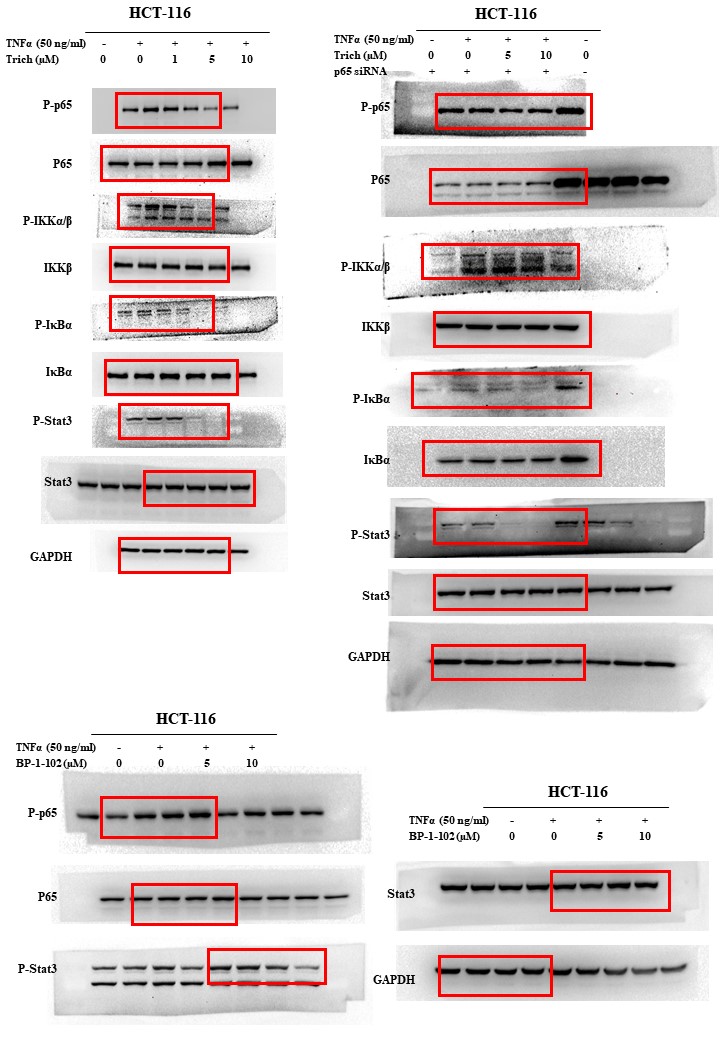

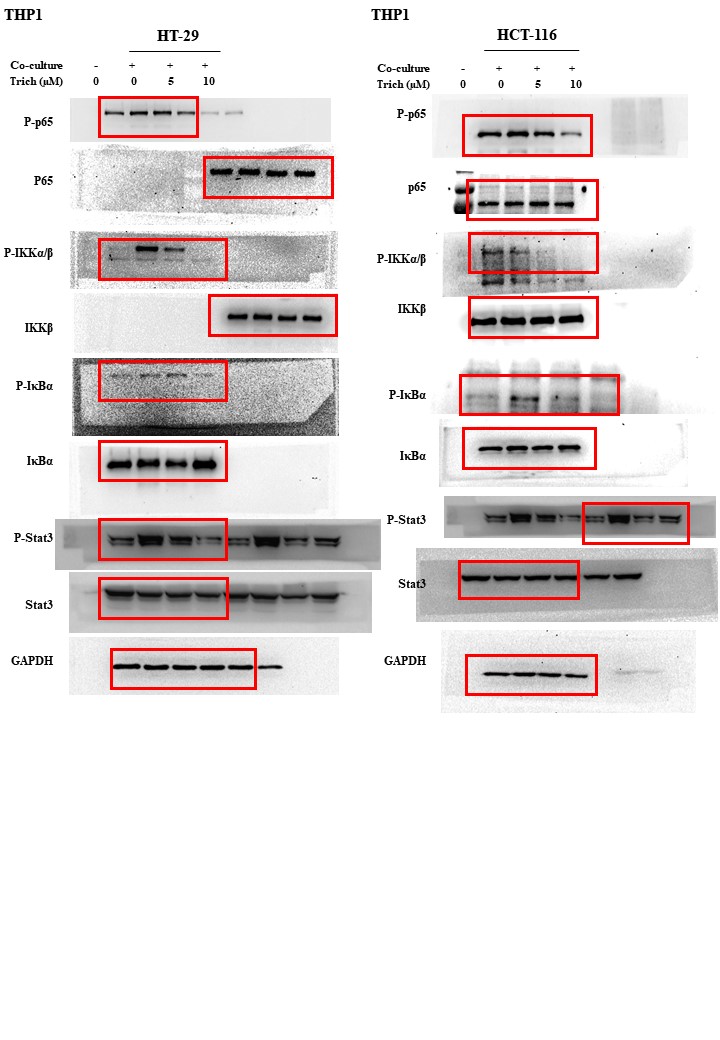

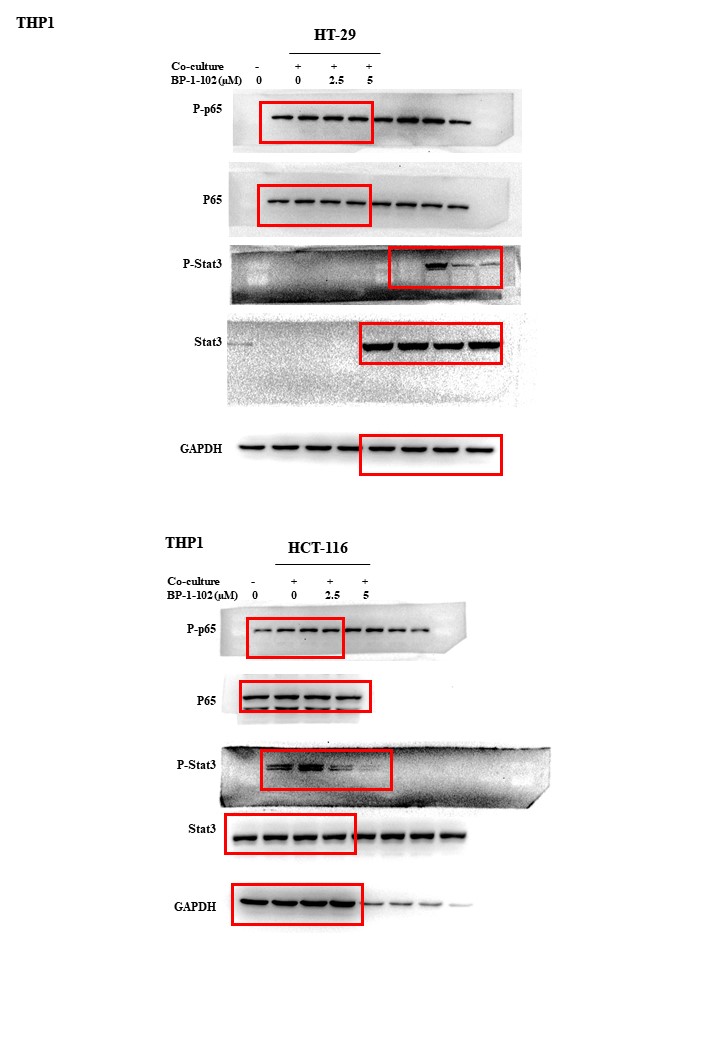

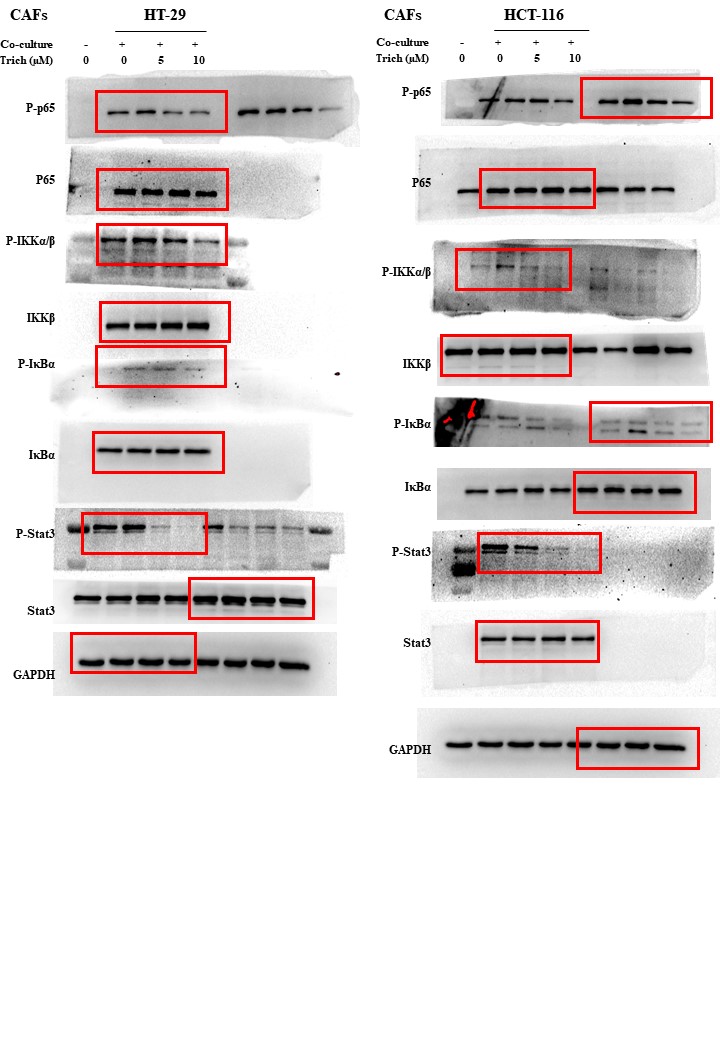

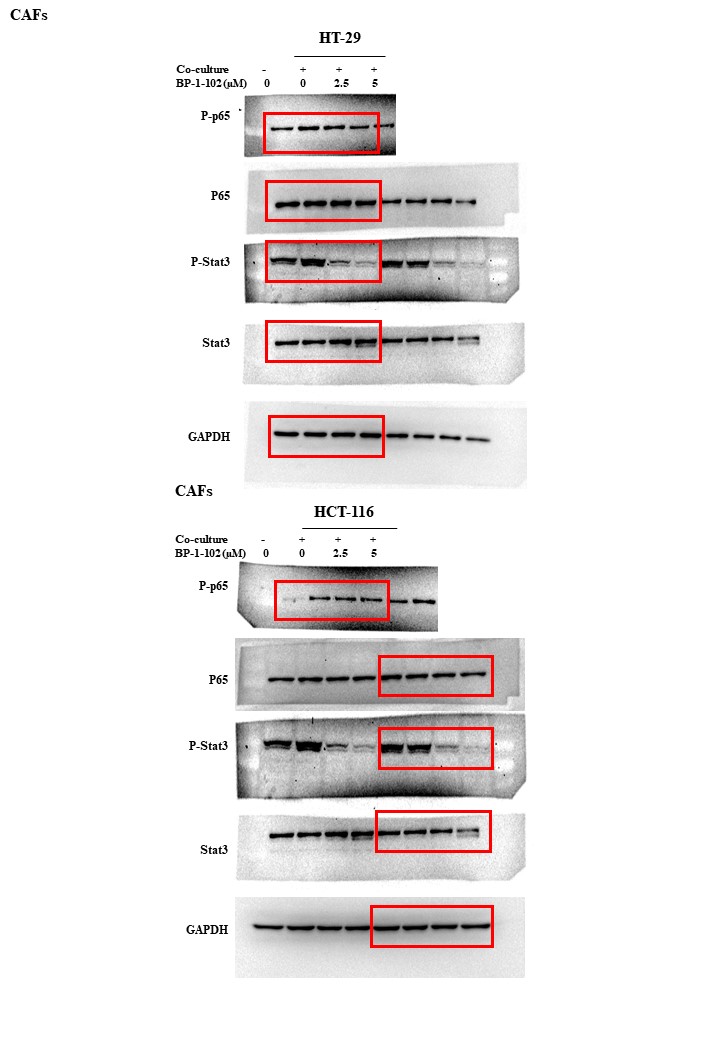

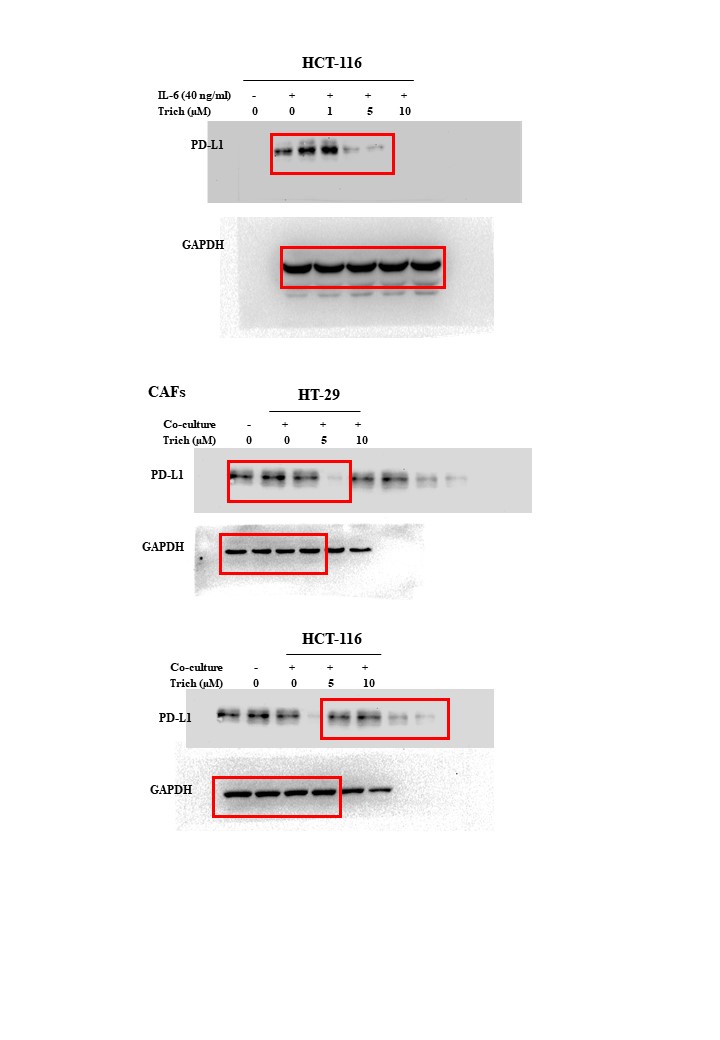

Supplement: Supplementary file 1 [file DataSheet_1.docx]
